# A Novel Bio-Adhesive Based on Chitosan-Polydopamine-Xanthan Gum for Glass, Cardboard and Textile Commodities

**DOI:** 10.3390/polym16131806

**Published:** 2024-06-26

**Authors:** Jessica Costa, Maria Camilla Baratto, Daniele Spinelli, Gemma Leone, Agnese Magnani, Rebecca Pogni

**Affiliations:** 1 Department of Biotechnology, Chemistry and Pharmacy, University of Siena, Via Aldo Moro 2, 53100 Siena, Italy; jessica.costa2@unisi.it (J.C.); mariacamilla.baratto@unisi.it (M.C.B.); gemma.leone@unisi.it (G.L.); agnese.magnani@unisi.it (A.M.); 2 Centre for Colloid and Surface Science (CSGI), Via della Lastruccia 3, 50019 Sesto Fiorentino, Italy; 3 Next Technology Tecnotessile, Via del Gelso 13, 59100 Prato, Italy; daniele.spinelli@tecnotex.it

**Keywords:** chitosan, polydopamine, xanthan gum, bio-based adhesive, antiflame properties

## Abstract

The escalating environmental concerns associated with petroleum-based adhesives have spurred an urgent need for sustainable alternatives. Chitosan, a natural polysaccharide, is a promising candidate; however, its limited water resistance hinders broader application. The aim of this study is to develop a new chitosan-based adhesive with improved properties. The polydopamine association with chitosan presents a significant increase in adhesiveness compared to pure chitosan. Polydopamine is synthesized by the enzymatic action of laccase from *Trametes versicolor* at pH = 4.5, in the absence or presence of chitosan. This pH facilitates chitosan’s solubility and the occurrence of catechol in its reduced form (pH < 5.5), thereby increasing the final adhesive properties. To further enhance the adhesive properties, various crosslinking agents were tested. A multi-technique approach was used for the characterization of formulations. The formulation based on 3% chitosan, 50% polydopamine, and 3% xanthan gum showed a spectacular increase in adhesive properties when tested on glass, cardboard and textile. This formulation increased water resistance, maintaining the adhesion of a sample soaked in water for up to 10 h. For cardboard and textile, material rapture occurred, in mechanical tests, prior to adhesive bond failure. Furthermore, all the samples showed antiflame properties, expanding the benefits of their use. Comparison with commercial glues confirms the remarkable adhesive properties of the new formulation.

## 1. Introduction

Nowadays, environmental issues have garnered increasing attention with a key concern being heavy reliance on petroleum resources. The irreversible effects of climate change underscore the urgent need for a swift transition from fossil-based to bio-based materials [1]. Adhesives play a pivotal role in everyday life, poly(vinyl acetate), epoxy, phenol-formaldehyde, and polyurethane derived from fossil-based feedstock, represent only a few examples. Numerous adhesives contain toxic substances that are harmful for the environment and human health [2,3]. Moreover, the flammability of most of them poses a significant hazard for fire incidents [4]. Hence, there is a growing interest in developing new bio-based and more sustainable adhesives [5,6]. Polysaccharides and lignin, in particular, play a significant role in the preparation of natural adhesives. They are described as natural mediators for surface adhesion in nature, often in conjunction with other compounds [7]. Recently, an adhesive based on starch and lignosulphonate crosslinked with laccase, has been proposed for the bonding of paper [8,9]. A new adhesive formed by epoxidized kraft lignin in the form of lignin nanoparticles and biocolloids has produced a strong adhesive for wood comparable to commercially available ones [10]. Among polysaccharides, chitosan (CHIT) stands out as a promising adhesive material [11,12]. Chitosan, the deacetylated form of chitin, is composed of (1 → 4)-D-glucosamine (GlcN) residues, with chitin, (1 → 4)-N-acetyl-D-glucosamine (GlcNAc), being one of the most abundant biopolymers globally, sourced from various natural materials such as the exoskeletons of crustaceans, insect cuticles, fungal cell walls, and algae [13]. Chitosan possesses important properties including biodegradability, biocompatibility, non-toxicity, antimicrobial activity, and adhesiveness [12]. Consequently, it finds wide application across various sectors including pharmaceuticals, food packaging, textiles, agriculture, cosmetics, and even as a support for enzymatic immobilization [14,15]. Chitosan is soluble in aqueous acidic conditions due to the protonation of primary amine groups, with a pKa around 6.5, and it can be dissolved in acetic acid to form a viscous solution. This solution solidifies into a strong adhesive bond through water loss via evaporation or absorption by porous adherent materials [2]. Despite its high cohesive strength, chitosan exhibits limited water resistance. To address this limitation, various molecules have been explored for adhesive applications, including crosslinking agents, which can reduce water accessibility within the network by promoting molecular entanglement through the formation of bridges between polymer chains [16]. Another approach involves hydrophobization, where hydrophobic substances are incorporated into polymer molecules through chemical grafting or suitable copolymerization procedures [17]. In alignment with the objective of developing bio-based materials, nature offers numerous examples of functional materials, with one of the most intriguing being the water-resistant adhesive used by marine organisms such as mussels. The strength of mussel adhesive is attributed to the presence of crosslinkers between polymer chains, a process termed “curing”, which is believed to require a catechol precursor, (e.g., 3,4-dihydroxyphenylalanine (DOPA)), and a catechol oxidase. The oxidation of DOPA leads to the formation of quinone, providing the moisture-resistance characteristic of mussel underwater adhesion [18]. A novel bio-based supramolecular adhesive, produced by the partial coordination of melevodopa functionalized castor oil and Fe^3+^ ions, has shown outstanding adhesive performance for multimaterials [19]. Inspired by nature, the use of 3,4-dihydroxyphenethylamine (dopamine) has been investigated to impart water-resistant adhesive properties to chitosan. Laccase from *Trametes versicolor* (*Tv*), an oxidative enzyme, serves as a catechol oxidase. The oxidation of dopamine (DA) leads to the formation of polydopamine (PDA), which exhibits great structural similarity and properties to melanin pigments. Melanin-like polymers are non-toxic and biocompatible, with their chemical structure based on catechol fractions being heterogeneous and dependent on polymerization conditions [20,21,22,23]. This study focuses on chitosan-based adhesive formulations incorporating chitosan-polydopamine and crosslinking agents synergistically, with the primary goal of enhancing adhesive strength, water resistance, and imparting flame-retardant characteristics [24]. To evaluate the effectiveness of the chitosan-based adhesive formulations, various materials were selected for testing. Glass was chosen to assess water resistance, and cardboard was selected due to its common use in packaging. Additionally, textile materials made with heat-pressed waste fibers were included to simulate scenarios for the development of interior design furniture, highlighting the importance of bio-based adhesives and flame-retardant properties in such applications. Mechanical properties were determined to identify the optimal formulation.

## 2. Materials and Methods

### 2.1. Materials

Chitosan (CHIT), from the shells of *Pandalus borealis* (cold-water shrimp), with a molecular weight ranging from 50,000 to 190,000 Da with a deacetylation degree of 75–85%, xanthan gum (XG), *β*-(1 → 4)-D-glucopyranose glucan backbone with side chains of (1 → 3)-α-D-mannopyranose-(2 → 1)-β-D-glucuronic acid-(4 → 1)-β-D-mannopyranose on alternating residues, from *Xanthomonas campestris*, dopamine hydrochloride ≥ 97.5 *w*/*w*% (DA), laccase from *Trametes versicolor* (*Tv*) (1.0 U/mg), phytic acid from rice 90–95 *w*/*w*% (PA), citric acid ≥ 99.5 *w*/*w*% (CA), glutaraldehyde 25 aqueous solution *w/v*% (GLU), trisodium trimetaphosphate ≥ 99.5 *w*/*w*% (TTMP), and genipin ≥ 98 *w*/*w*% (GEN), were obtained from Sigma-Aldrich (St. Louis, MO, USA) and used without further purification. Glass slides and common cardboard were supplied by the Aiesi hospital service (Italy). Textile material was provided by Next Technology Tecnotessile (NTT-Prato-Italy) and produced using air-laid technology followed by thermal compression at 150 °C. The textile includes a fiber blend of polyester and cotton 70/30 (*w*/*w*%) (2 mm thickness; 1000 g/m^2^).

### 2.2. Synthesis and Characterization of Polydopamine

Polydopamine was synthesized using an enzymatic method through the oxidative action of laccase. A total of 0.6 mg of dopamine hydrochloride (1 mM) and 2.1 mg of laccase (1.0 U/mg–0.01 mM) were solubilized in 3 mL of 2% acetic acid. The molar ratio of enzyme:substrate was 1:100. The reaction mixture was left to react in a shaker at 150 rpm, under 25 °C for 24 h. Another approach for synthesizing polydopamine in the presence of chitosan was evaluated. First, 60 mg of chitosan was solubilized in 3 mL of 2% acetic acid at 60 °C, and then, 0.6 mg of dopamine hydrochloride and 2.1 mg of laccase were added. The reaction was left to react in a shaker at 150 rpm, under 25 °C for 24 h. The synthesis was monitored using a UV-Visible spectrometer (Lambda 900/Perkin Elmer Instrument—Norwalk, CT, USA). When the reaction was concluded, the samples were dried under nitrogen flux and analyzed using Fourier transform infrared spectroscopy (FT-IR—Agilent Technologies Cary 630 FT-IR—Santa Clara, CA, USA). 

### 2.3. Samples Preparation and Characterization and Water Resistance Test 

**Scheme 1:**
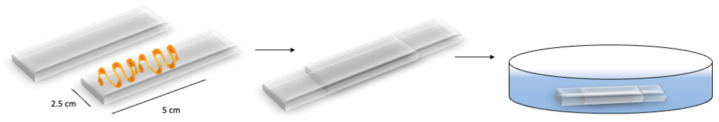
Procedure for water resistance test.

Aqueous solutions of chitosan (1%, 2% and 3%) were prepared by adding 10, 20 and 30 mg of chitosan, respectively, into 1 mL of 2% acetic acid at 60 °C for 10 min under magnetic stirring. The synthesized polydopamine was added at different concentrations to the chitosan solutions. The following formulations were prepared and tested on glass, cardboard and textile (Table 1):

The best chitosan and chitosan-polydopamine samples were selected based on water resistance tests, as described below and shown in Figure S1. The water resistance properties were tested using two slides of glass (surface 2.5 × 5 cm) covered with 0.019 g/cm^2^ of adhesive, overlapped and pressed with fingers at room temperature. The glass surfaces were previously washed with ethyl alcohol to remove any trace of impurities. The samples were allowed to dry for one week in air. Subsequently, to test water resistance, the samples were soaked in 50 mL of distilled H_2_O (T = 25 °C) in a Petri dish and monitored over time [11]. The test was concluded when the glass slides lost adhesion. All tests were conducted in triplicate. In Scheme 1, the procedure for the water resistance test is summarized. 

On the basis of the water resistance test, a 3% chitosan—50% polydopamine solution was selected and used, as basic preparation, before the addition of different percentages of crosslinking agents. The density of the chosen bio-adhesive formulation, based on 3% chitosan, 50% polydopamine and 3% xanthan gum, was measured at 25 °C using a 5mL pycnometer (nach Gay-Lussac Blaubrand mit Einzelzertifikat, justiert, Boro 3.3, ISO 9001-14001 certified [25]) from Brand Gmbh + Co KG Postfach, Wertheim/Main, Germany. After preparation, the formulation was placed in a pycnometer up to a volume of 5 cm^3^. The density (d) was calculated by subtracting the weight of the empty pycnometer from the weight of the pycnometer with the adhesive. This weight, corresponding to the mass of the adhesive (M), was then divided by the volume (V). The density is given by the formula d (g/cm^3^) = M (g)/V (cm^3^). The pH of the chitosan, polydopamine and xanthan gum adhesive formulation was measured using a manual pHmeter FiveGo™ from Mettler Toledo AG, Columbus, OH, USA, Analytical CH-8603 Schwerzenbach, Zurich, Switzerland. The viscosity of the formulation was analyzed using a Discovery Hybrid Rheometer 2 (TA Instruments, Leatherhead, UK) with plate–plate stainless-steel geometry. Data analysis was performed with TRIOS v.4.1.1.33073 software. The procedure involved a peak hold step at a constant shear rate of 100 s^−1^ and a temperature of 25 °C, maintained until the material reached its maximum viscosity and then a plateau, to identify the gelation time. For comparison, the same experimental tests, on glass, cardboard and textile, were carried out using two commercial glues: UHU glue stick, made in Germany (Uhu GmbH & Co. KG, Herrmannstr. 7, Berlin, Germany, D-77815) and hot glue from Tiger manufactured by Zebra A/S Strandgade 71, DK-1401 Copenhagen, Denmark. 

### 2.4. Dynamic Light Scattering (DLS) Analysis

Dynamic light scattering (DLS) measurements were performed using a Zetasizer NanoZS90 instrument (Malvern Instrument Ltd., Worcestershire, UK). The experiments were performed on the following samples: chitosan 3%, polydopamine, chitosan-polydopamine, chitosan-polydopamine-trisodium trimetaphosphate 1%, chitosan-polydopamine-genipin 1% and chitosan-polydopamine-xanthan gum 1%. The samples were diluted with 2% acetic acid maintaining the acidic pH. The DLS analysis was used to evaluate the variation in particle charge and size.

### 2.5. Flame Retardancy Properties 

The use of chitosan, as a flame retardant, is quite new and represents a green and sustainable approach to confer antiflame properties to different polymers [24]. Furthermore, polydopamine with its nitrogen content can enhance this aspect. Preliminary experiments to test flame retardance were performed through fire testing and thermogravimetric analysis (TGA). Fire testing was carried out by covering the surface of the cardboard and textile with adhesive, and when dried, the samples were burned. Thermal stability was analyzed via thermogravimetry (SDT-Q600—TA Instruments). The temperature range was 25–900 °C under a nitrogen atmosphere. The samples analyzed included textile alone, textile covered with chitosan and chitosan-polydopamine. Prior to the analysis, the samples were dried. 

### 2.6. Mechanical Properties 

The adhesive’s adhesion properties were tested through the determination of tensile lap-shear strength according to UNI EN 1465 [26]. The analysis was conducted using two dynamometers, Instron Model 34SC-1 and 3369 Single Column. Tests were performed for cardboard and textile and the adhesive formulations analyzed were chitosan 3%, chitosan-polydopamine 50% and chitosan-polydopamine 50% and xanthan gum 3%. Additionally, a 3% xanthan gum sample was tested for comparison purposes. The mechanical tests were performed using a crosshead speed equal to 30 mm/min. The same parameters were used to test the mechanical properties of commercial glues (see Table S1).

## 3. Results and Discussion

**Figure 1:**
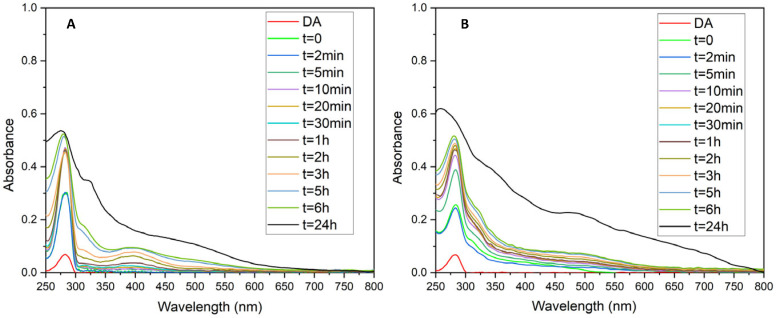
UV-visible spectrophotometric analysis for monitoring the reaction time of (**A**) polydopamine synthesis and (**B**) polydopamine synthesis in presence of chitosan. The analysis was performed at room temperature and monitored at the starting time (t = 0), after 2 min, 5 min, 10 min, 20 min, 30 min, 1 h, 2 h, 3 h, 5 h, 6 h and 24 h. In Figure 1, the spectrum of the precursor dopamine (DA) is reported for comparison.

The adhesive properties of chitosan and the chitosan-polydopamine system are well-documented, primarily for biomedical applications [27,28]. In this study, the chitosan-polydopamine system was tested in the presence of other crosslinking agents with the aim of enhancing the bio-adhesive properties for its use in common goods to replace conventional adhesives. In this context, the formulations were characterized and tested on glass, cardboard, and textile, and their mechanical and flame-retardant properties were analyzed. The preparation of the formulations began with the synthesis and characterization of polydopamine. Polydopamine is typically produced by the spontaneous oxidation of dopamine in basic conditions. However, this method is unsuitable for our system due to the specific solubility of chitosan in acidic media. Therefore, polydopamine synthesis was carried out using the oxidative enzyme laccase, enabling synthesis at pH = 4.5 [29]. Two different synthetic approaches, with and without chitosan, were employed: *intrinsic* and *extrinsic*. Both reactions were monitored by recording UV-visible spectra over time (Figure 1). Initially, the spectra exhibited the characteristic absorption peak of dopamine at a wavelength of 280 nm. In the first few minutes of the reaction in the *extrinsic* synthetic method (without chitosan) (Figure 1A), the formation of intermediate quinone, dopaminoquinone, was observed at 390 nm. This absorption peak was not observed in the *intrinsic* synthesis (Figure 1B), likely due to its rapid conversion to dopaminochrome. The difference between the synthesis for both methods (Figure 1A,B) suggests that the polymerization reaction of polydopamine is altered in the presence of chitosan. Two theories have been reported in the literature: the first suggests that the amino group of chitosan can react with dopaminoquinone to form Michael’s-type adducts, competing with the intramolecular reaction forming dopaminochrome. The formation of Michael’s adducts between quinones and amines has been observed in a region between around 340 and 400 nm. The second theory proposes that dopaminoquinone and/or dopaminochrome may undergo Schiff-base formation with chitosan’s amino groups [11]. Overall, polydopamine synthesized by the *extrinsic* and *intrinsic* methods exhibited similar UV-visible spectra, with a monotonic increase in absorbance at lower wavelengths characteristic of melanin-like polymer when the reaction was completed [20]. The synthesized polydopamine was further characterized by electron paramagnetic resonance (EPR) spectroscopy. In Figure S2A, the characteristic EPR spectrum of polydopamine is depicted, showing the persistent paramagnetism of the sample. The EPR spectrum exhibited a slightly asymmetrical signal characterized by g = 2.0038 ± 0.0001.

**Figure 2:**
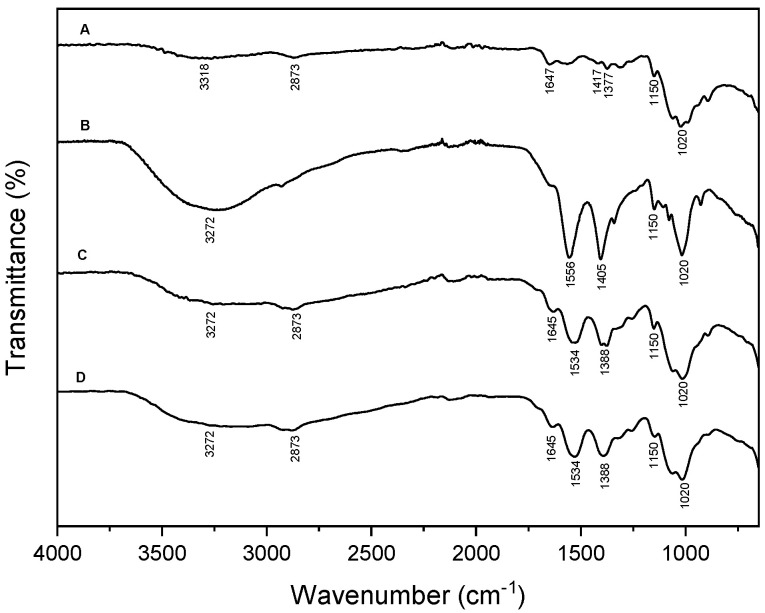
FT-IR spectra of: (**A**) chitosan, (**B**) polydopamine, (**C**) chitosan-polydopamine *extrinsic* and (**D**) chitosan-polydopamine *intrinsic*. The experiments were performed on powders and at room temperature.

A further investigation was carried out using FT-IR spectroscopy. In Figure 2, the spectra of polydopamine synthesized *extrinsically* (C) and *intrinsically* (D) are compared with the spectra of chitosan (A) and polydopamine (B). The analysis of the functional groups by FT-IR confirms the polymerization process occurred. The spectrum of chitosan (Figure 2A) exhibited absorption bands attributed to -OH and -NH stretching at 3318 cm^−1^. Additionally, the stretching vibration band of the C-H bond characteristic of this polysaccharide was observed at 2873 cm^−1^, while the peak at 1647 cm^−1^ corresponds to -C=O and the absorptions at 1417 and 1377 cm^−1^ are assigned to the hydroxyl groups of chitosan. Furthermore, the band observed at 1150 cm^−1^ resulted from the stretching vibration of C-O groups and the band at 1020 cm^−1^ can be attributed to the stretching vibration of -C-O-C- groups present in polysaccharides [30,31]. The polydopamine spectrum depicted in Figure 2B reveals distinctive features, including a large band at 3272 cm^−1^ attributed to -OH and -NH stretching, alongside peaks at 1556 cm^−1^ and 1405 cm^−1^ corresponding to the C=N and C-N-C stretching vibration, respectively. Furthermore, the presence of -C-O-C- stretches resulting from a laccase-catalyzed reaction is evidenced by two peaks at 1150 and 1020 cm^−1^, indicating potential ether bond linkage between dopamine units [29,32,33]. The spectra of polydopamine synthesized *extrinsically* (C) and added to chitosan, and *intrinsically* (D) (polydopamine-chitosan), are characterized by similar absorption bands. The -OH and -NH stretching was observed at 3272 cm^−1^, the asymmetric and symmetric stretching of -CH was represented by a band around 2900 cm^−1^, the -C=O stretching of amide I was observed at 1645 cm^−1^ [34,35].

The water resistance property of chitosan-based adhesive formulations was tested by soaking glass samples in water. Initially, different concentrations of chitosan were analyzed (1%, 2%, and 3%). The best result was obtained for chitosan at 3%. Subsequently, the polydopamine synthesized using *extrinsic* and *intrinsic* methods was compared. The *extrinsic* synthesis showed higher water resistance, and this formulation was chosen for further experiments. For all percentages of polydopamine added to chitosan, a clear increase in adhesiveness compared to pure chitosan particles was observed (See Figure S1). The larger dimensions of polydopamine in *extrinsic* synthesis stimulated crosslinking, resulting in stronger adhesive bonds. Despite the potential hindrance of adhesive properties due to the consumption of catechol groups to establish internal bonds, a good balance was achieved with the presence of chitosan groups and a proper dispersion of polydopamine in the matrix. In contrast, *intrinsic* synthesis involving dopamine as a precursor, a smaller molecule, could react with multiple amine groups of chitosan, potentially decreasing adhesive properties [2]. For the polydopamine-chitosan system, the most significant increase in water resistance was observed with 50% polydopamine (Figure S1) [36,37,38].

**Figure 3:**
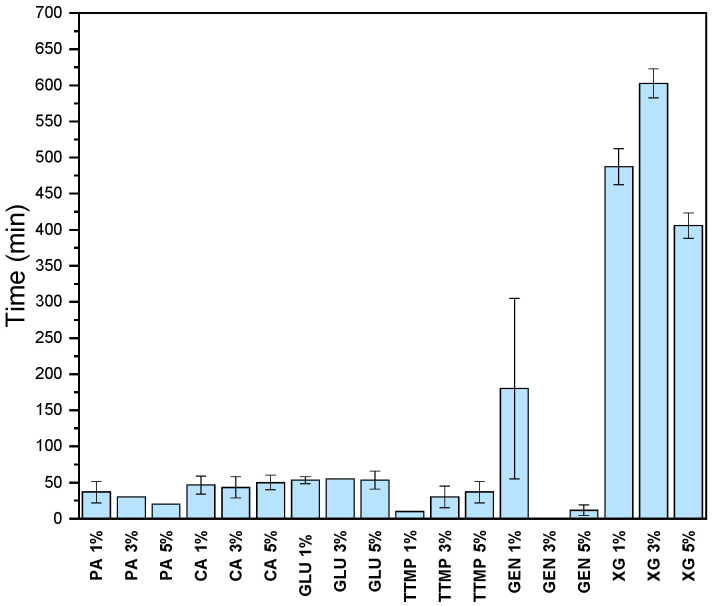
Water resistance analysis. Chitosan-polydopamine and different concentration of crosslinkers (1%, 3%, 5%). All the analyses were performed in triplicate.

Given the basic formulation of a 3% chitosan solution with 50% polydopamine (relative to the amount of chitosan), the addition of other crosslinking agents, with different chemical characteristics, was tested to evaluate if an increase in adhesiveness was achieved. Depending on the nature of the crosslinkers, interactions forming the network could be covalent, ionic, or both [39]. In this context, phytic acid (PA), citric acid (CA), glutaraldehyde (GLU), trisodium trimetaphosphate (TTMP), genipin (GEN), and xanthan gum (XG) were selected as crosslinkers. These molecules have diverse structures and mechanisms of interaction with chitosan. Phytic acid, obtained from plant tissues, has raised interest for its fire-retardant properties [39,40,41]. Phytic acid, as well as trisodium trimetaphosphate, are polyphosphate compounds suitable as crosslinking agents for chitosan through electrostatic interactions between phosphate and ammonium groups. However, this mechanism could result in charge neutralization of the polymer chain. Indeed, a milky suspension was generated upon adding phytic acid to the chitosan solution, reducing its adhesive properties and thus decreasing water resistance (Figure 3) [42,43]. Another electrostatic interaction was studied using a polyanionic compound, citric acid (CA). It was demonstrated that the presence of citric acid improved mechanical strength; however, the ionic interactions between the negative charge of citric acid and the positive charge of chitosan decreased the adhesion properties of the formulation [38,44].

**Figure 4:**
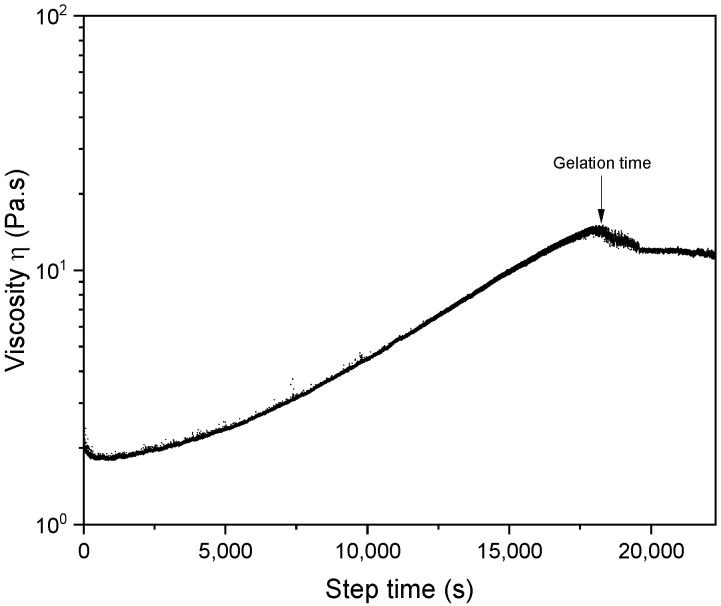
Graph of gelation time measurement performed by rheometer at 25 °C on 3% chitosan, 50% polydopamine and 3% xanthan gum formulation.

Based on the data obtained, ionic crosslinking agents worsened the water resistance properties of the adhesive. Consequently, the use of covalent crosslinkers was explored. Glutaraldehyde and the bioresource-based crosslinker genipin from geniposide in *Gardenia Jaminoides Ellis* fruits were tested [45]. It was reported that using glutaraldehyde as a crosslinker produces a more hydrophobic structure in chitosan [46,47]. However, in our case, the results obtained for both crosslinking agents, except in the case of genipin at 1%, did not significantly change the water resistance properties [48]. These experiments showed that neither covalent nor ionic crosslinking agents added new properties to the chitosan-polydopamine formulation. On the other hand, interesting results were obtained with the addition of small percentages of xanthan gum, a microbial exopolysaccharide with a cellulosic backbone with two mannose and one glucuronic acid side chain on every second glucose residue. It is an anionic polyelectrolyte with a molecular weight that can reach up to 6 million Daltons, making it possible to create extremely viscous solutions at very low concentrations [49]. In this case, a spectacular increase in water resistance was achieved with xanthan gum and the chitosan-polydopamine formulation (Figure 3), where the addition of 3% xanthan gum maintained the adhesion of the sample soaked in water for up to 10 h. The chosen adhesive formulation, composed of 3% chitosan, 50% polydopamine and 3% xanthan gum, exhibited a density of 1.19 ± 0.22 g/cm^3^ and pH= 3.82. The formulation was analyzed in terms of viscosity and gelation time. Figure 4 shows the viscosity spectrum over time, with gelation time observed at 18,042.6 s (5 h), corresponding to a viscosity of 14.43 Pa⋅s. 

For comparison, commercial glues were tested and the results are shown in Table S1. Concerning the water resistance test, both commercial glues demonstrated a very low or inexistent adhesiveness, the UHU glue stick showed water resistance for 69 ± 20 min, whereas the Tiger hot glue could not be tested due to its low adhesive properties on glass. In the mechanical tests, both of them showed worse results for cardboard and textile with Tiger hot glue that was not suited for cardboard. These results highlight the excellent properties of the new CHIT-PDA-XG formulation, as demonstrated by the investigation of adhesive performance on glass, cardboard, and textiles reported in Figure S3.

**Figure 5:**
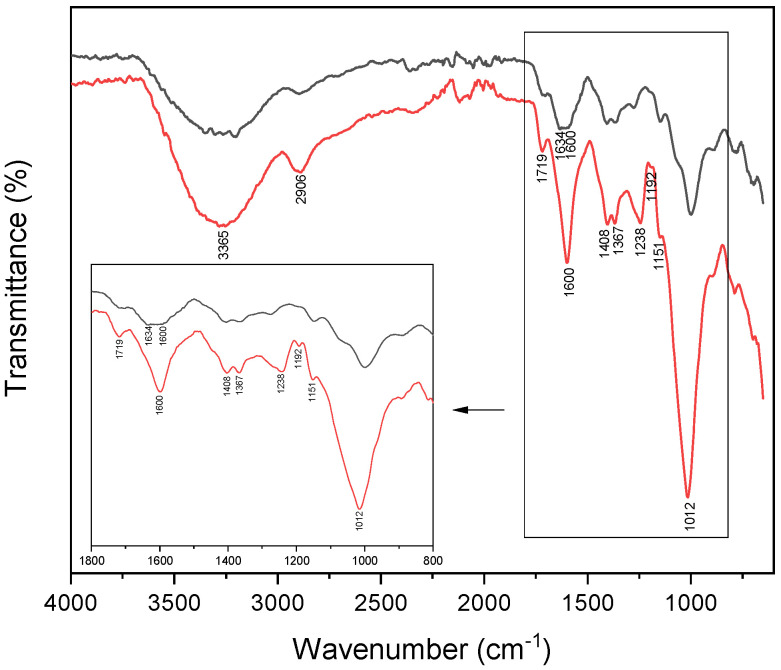
FT-IR spectra of xanthan gum alone (red line) and xanthan gum after reaction with laccase (black line). In the inset, the region from 1800 to 800 cm^−1^ is highlighted. The sample of oxidized xanthan gum was prepared maintaining the same ratios used in the final chitosan-polydopamine-xanthan gum formulation. The analysis was performed on powders at room temperature.

The outmost adhesive performance of the sample containing xanthan gum, might be attributed to the negative charges of the biopolymer, observed in the pH range of 2.4–6.3 [50], stemming from the presence of carboxylic groups. These groups form a polyelectrolyte complex through reversible ionic linkages with the protonated free amino groups of chitosan [51]. Regarding surface charge, xanthan gum exhibits similar behavior to the other crosslinkers (see Figure 5), suggesting that the enhancement in adhesive properties might be linked to the presence of laccase in the chitosan-polydopamine formulation. Laccase can oxidize xanthan gum, leading to the cleavage of C-C bonds between (-CHOH)_2_ groups to form dialdehydes. This not only decreases the adhesive’s hydrophilicity but also offers a route for covalent interaction with the substrate, thereby improving water resistance. To test this assumption, the FT-IR spectrum of xanthan gum (red line) is compared with that of xanthan gum oxidized by laccase (black line) in Figure 5. In the inset, the region 1800–800 cm^−1^ is highlighted. The absorption band at 1600 cm^−1^ assigned to -COO^−^ is evident in the xanthan gum spectrum, but after oxidation, it is split into two bands where the one at 1634 cm^−1^ is assigned to the presence of -CHO groups. The region around 1200 cm^−1^ assigned to -C-O-C ether is modified in the oxidized xanthan gum due to the opening of the saccharide ring [52]. Furthermore, the combination of oxidized xanthan gum with chitosan should improve the adhesion properties due to crosslinking of the aldehydes with the amino groups to form an imine linkage (Schiff base) [52].

**Figure 6:**
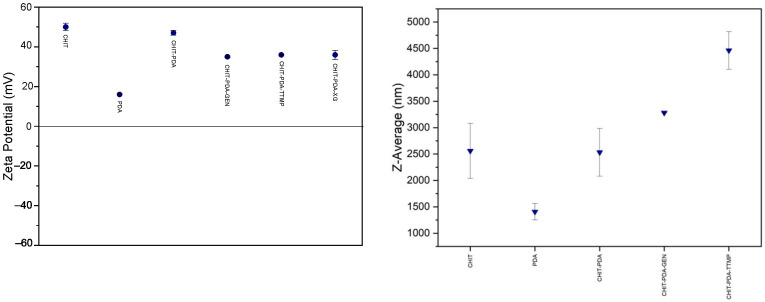
Dynamic light scattering (DLS) analysis. On the left, the zeta potential of chitosan 3% (CHIT), polydopamine (PDA), the adhesive formulation of chitosan and 50% of polydopamine (CHIT-PDA), the adhesive formulation of chitosan and 50% of polydopamine in the presence of 1% of crosslinkers genepin, trisodium trimetaphospate and xanthan gum (CHIT-PDA-GEN, CHIT-PDA-TTMP, CHIT-PDA-XG, respectively) are reported. The samples were diluted with 2% acetic acid. On the right, the particle size of chitosan 3% adhesive (CHIT), polydopamine (PDA), the adhesive formulation of chitosan and 50% of polydopamine (CHIT-PDA) and the adhesive formulation of chitosan and 50% of polydopamine in presence of 1% of crosslinkers genepin and trisodium trimetaphospate (CHIT-PDA-GEN and CHIT-PDA-TTMP, respectively) is reported.

Dynamic light scattering (DLS) analysis represents another important step in the characterization of the developed formulations. The zeta potential and size values for both chitosan and polydopamine polymers, as well as the developed formulations, are shown in Figure 6 and summarized in Table 2 along with the polydispersity index (PDI). The native polymers and all the formulations (except for chitosan-polydopamine-xanthan gum, the particle size of which is too high to be analyzed by DLS) exhibit monodisperse systems, as demonstrated by the polydispersity index values. Both chitosan and polydopamine show a positive zeta potential due to the positive charge of protonated amino groups. Even with the addition of both covalent and ionic crosslinkers, the positive value of chitosan remains significantly high, consistently maintained above 30.0–35.0 mV, demonstrating the good colloidal stability of the systems. Addition of crosslinkers reasonably increases the particle size, as demonstrated by the values reported in Table 2, with higher dimensions reached with the ionic one, trisodium trimetaphosphate. The addition of xanthan gum, due to the large size of the polymer, renders the system unanalyzable in terms of size with the DLS technique. However, its colloidal stability is still ensured by the zeta potential value, which remains higher than 35.0 mV.

**Figure 7:**
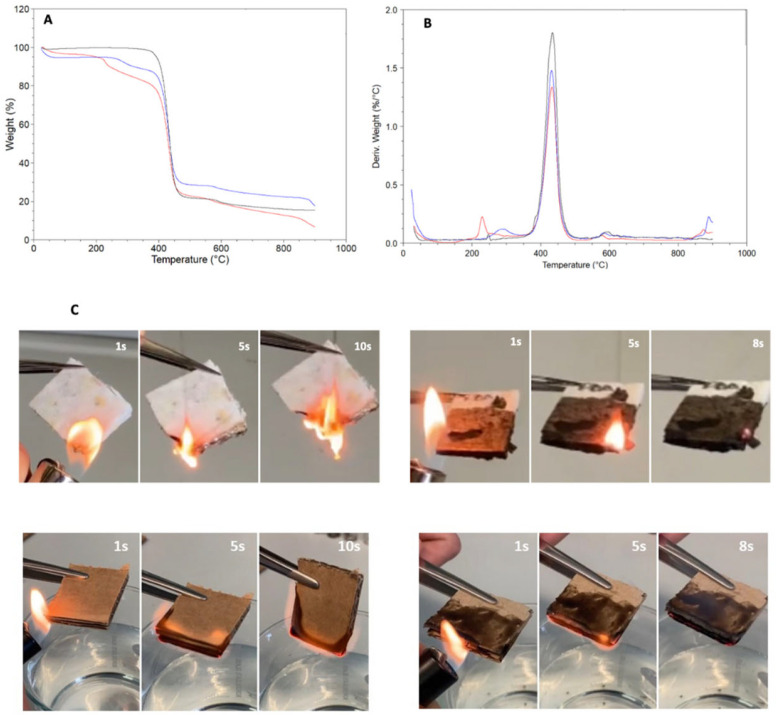
Thermogravimetric analysis (TGA) curves (**A**) and derivative thermogravimetric (DTG) curves (**B**) of textile and textile covered by chitosan and chitosan-polydopamine, measured under nitrogen. Black line: textile without adhesive; blue line: textile covered by chitosan and red line: textile covered by chitosan-polydopamine adhesive. (**C**) The textile and cardboard samples without adhesive are reported on the left (top and bottom, respectively). The samples covered by the adhesive are reported on the right (top and bottom, respectively). The black color of the adhesive is due to the presence of polydopamine.

The thermal behavior was analyzed by conducting thermogravimetric analysis on textile samples (Figure 7A,B), with the black line representing untreated textile, the blue line representing textile covered with chitosan, and the red line representing textile covered with chitosan-polydopamine. To demonstrate the interaction between the textile and the chitosan-polydopamine formulation, electron paramagnetic resonance (EPR) spectra were recorded. In Figure S2B, the black line represents a textile sample with no radical signal, and the red line indicates the presence of a radical signal due to the presence of polydopamine on the textile sample. The initial decomposition observed from 40 °C to 200 °C in textile covered by chitosan and chitosan-polydopamine was attributed to water loss due to the polymer’s hygroscopic properties, with an estimated weight loss of 5%. The second decomposition, occurring from 200 °C to 400 °C, was attributed to the rupture of C-C and C-O bonds of polymeric chains, resulting in a weight loss of 10% and 15% for textiles covered by chitosan and chitosan-polydopamine, respectively. The maximum degradation temperature was observed at 440 °C, with weight losses of 75% for untreated textile, 55% for textile covered by chitosan, and 53% for textile covered by chitosan-polydopamine. The differences in degradation weight loss are illustrated in Figure 7B, where the derivative weight (%/°C) is shown. The higher peak corresponds to untreated textile, the medium peak to textile covered by chitosan, and the lower peak to textile covered by chitosan-polydopamine. Additionally, the percentage of residues at 800 °C was 25% for textile covered by chitosan, 19% for untreated textile, and 12% for textile covered by chitosan-polydopamine. The preliminary results of the flammability test for coated and uncoated textiles and cardboard are reported in Figure 7C. The uncoated samples burned rapidly, whereas limited flame propagation was observed for the coated samples, with the flame extinguishing after 8 s. This behavior was attributed to the presence of nitrogen in the chitosan and polydopamine structures. In combustion reactions, nitrogen absorbs heat rather than releasing it, as the combustion of nitrogen is an endothermic process. All adhesive formulations demonstrated antiflame properties [24].

The mechanical properties of the adhesive formulations were tested according to UNI EN 1465 for determining the tensile lap-shear strength of bonds. The adhesive properties of the novel chitosan-polydopamine-xanthan gum formulation demonstrate remarkable efficacy. Its adhesion force surpasses the tensile strength of the materials, resulting in material fracture before adhesive bond failure (refer to Figure S4). Table 3 presents a comparison of tensile strengths among different samples: chitosan, chitosan-polydopamine, and chitosan-polydopamine-xanthan gum. The chitosan adhesive exhibited a tensile strength of 0.298 (N/mm^2^) for cardboard and 0.240 (N/mm^2^) for textile. The chitosan-polydopamine adhesive yielded a tensile strength of 0.228 (N/mm^2^) for cardboard and 0.198 (N/mm^2^) for textile. However, with the addition of xanthan gum, the adhesive bond demonstrated a stronger force than the tensile forces for both materials, highlighting the excellent adhesiveness of the formulation. Additionally, as part of a control test, the adhesive behavior of xanthan gum alone was examined, revealing a tensile strength of 0.143 (N/mm^2^). The synergistic effect of chitosan combined with polydopamine and xanthan gum represents an outstanding adhesive formulation for all the bonded materials and, above all, a higher adhesiveness compared to analyzed commercial glues. 

## 4. Conclusions

Development of potential bio-based adhesives has garnered great interest among consumers due to the excessive use of synthetic adhesives, their toxicity, and the associated environmental problems. Chitosan has emerged as a good candidate for potential natural adhesive use, but its poor water resistance has hindered its application. The incorporation of polydopamine, synthesized by *Tv* laccase in the absence and presence of chitosan, leads to an increase in water resistance. Furthermore, other crosslinking agents were added to enhance the adhesiveness of the formulations. In the presence of xanthan gum alone, a spectacular increase in adhesive properties was detected. This is probably due to the formation of aldehyde groups through the oxidative activity of laccase on xanthan gum. A formulation based on 3% chitosan, 50% polydopamine and 3% xanthan gum is able to bond different materials like glass, cardboard and textile. It shows excellent water resistance (bonding failure until 10 h) and high tensile strength, resulting in material fracture prior to adhesive bond failure for cardboard and textile. Furthermore, preliminary results show the antiflame capacity of the formulation. Its remarkable adhesive properties, when compared with commercial glues, open new avenues of application.

## Figures and Tables

**Table 1:** Adhesive formulations at different concentrations of chitosan, PDA and crosslinkers.

Adhesive Formulations							
1	CHIT 1%			14	CHIT 3%	PDA extrinsic 50%	CA 5%
2	CHIT 2%			15	CHIT 3%	PDA extrinsic 50%	GLU 1%
3	CHIT 3%			16	CHIT 3%	PDA extrinsic 50%	GLU 3%
4	CHIT 3%	PDA extrinsic 5%		17	CHIT 3%	PDA extrinsic 50%	GLU 5%
5	CHIT 3%	PDA extrinsic 10%		18	CHIT 3%	PDA extrinsic 50%	TTMP 1%
6	CHIT 3%	PDA extrinsic 20%		19	CHIT 3%	PDA extrinsic 50%	TTMP 3%
7	CHIT 3%	PDA extrinsic 30%		20	CHIT 3%	PDA extrinsic 50%	TTMP 5%
8	CHIT 3%	PDA extrinsic 50%		21	CHIT 3%	PDA extrinsic 50%	GEN 1%
9	CHIT 3%	PDA extrinsic 50%	PA 1%	22	CHIT 3%	PDA extrinsic 50%	GEN 3%
10	CHIT 3%	PDA extrinsic 50%	PA 3%	23	CHIT 3%	PDA extrinsic 50%	GEN 5%
11	CHIT 3%	PDA extrinsic 50%	PA 5%	24	CHIT 3%	PDA extrinsic 50%	XG 1%
12	CHIT 3%	PDA extrinsic 50%	CA 1%	25	CHIT 3%	PDA extrinsic 50%	XG 3%
13	CHIT 3%	PDA extrinsic 50%	CA 3%	26	CHIT 3%	PDA extrinsic 50%	XG 5%

**Table 2:** Size and zeta potential data for both chitosan and polydopamine polymers and all the developed formulations.

Native Polymers/Network Formulations	Zeta Potential (mV)	Z-Average (nm)	PDI
CHIT	50 ± 2	2560 ± 521	0.95 ± 0.07
PDA	15.8 ± 0.3	1408 ± 155	0.5 ± 0.1
CHIT-PDA	47 ± 4	2533 ± 451	0.8 ± 0.2
CHIT-PDA-GEN	34.9 ± 0.4	3283 ± 237	0.69 ± 0.09
CHIT-PDA-TTMP	35.7 ± 0.2	4463 ± 357	0.69 ± 0.01
CHIT-PDA-XG	36 ± 2	n.d.	n.d.

**Table 3:** Tensile strength of chitosan (CHIT), chitosan-polydopamine (CHIT-PDA) and chitosan-polydopamine-xanthan gum (CHIT-PDA-XG) formulations on textile and carboard. The analysis was performed according to UNI EN 1465 for the determination of tensile lap-shear strength bonded. * Tensile strength is determined by the ratio of the maximum force (Fmax) to the cross-section area (Section).

		Section (mm^2^)	Fmax (N)	Tensile Strength * (N/mm^2^)
Cardboard	CHIT	1680	501.9	0.298
	CHIT-PDA	1710	390.4	0.228
	CHIT-PDA-XG	3150	999.2	n.d.
Textile	CHIT	1800	431.9	0.240
	CHIT-PDA	1800	356.6	0.198
	CHIT-PDA/XG	3501	665.1	n.d.

## Data Availability

Data are contained within the article.

## Supplementary Materials

The following supporting information can be downloaded at: https://www.mdpi.com/article/10.3390/polym16131806/s1, Figure S1: Water Resistance Analysis: Different concentrations of chitosan solution (1%, 2%, and 3%). The chitosan solution (3%) was selected, and various concentrations of polydopamine (5%, 10%, 30%, and 50%—relative to the amount of chitosan) were added. All samples were tested in triplicate. Figure S2: Continuous Wave Electron Paramagnetic Resonance Spectroscopy (CW-EPR) measurements at X-band (ν = 9.87 GHz). (A) Polydopamine spectrum (B) Adhesive formulation in textile. black line: textile without adhesive, red line: textile covered by chitosan/polydopamine adhesive. The analysis was performed at room temperature. Figure S3: Investigation of adhesive formulations on their performance onto glass (water bonding resistance), cardboard and textile (tensile strength). The samples compared include: 3% chitosan, 3% chitosan with 50% extrinsic polydopamine, 3% chitosan with 50% extrinsic polydopamine and 3% xanthan gum, commercial glue stick, and commercial hot glue. Figure S4: Photos taken during the mechanical testing of the chitosan/polydopamine/xanthan gum adhesive in cardboard (on the left) and textile (on the right). Table S1: Results of mechanical tests performed on two commercial glues.

## References

[1] Yang L., Wang X., Dai M., Chen B., Qiao Y., Deng H., Zhang D., Zhang Y., Villas Bôas de Almeida C.M., Chiu A.S.F. (2021). Shifting from fossil-based economy to bio-based economy: Status quo, challenges, and prospects. Energy.

[2] Patel A.K. (2015). Chitosan: Emergence as potent candidate for green adhesive market. Biochem. Eng. J..

[3] Kozicki M., Guzik K. (2021). Comparison of VOC Emissions Produced by Different Types of Adhesives Based on Test Chambers. Materials.

[4] Saba N., Jawaid M., Paridah M.T., Al-othman O.Y. (2016). A review on flammability of epoxy polymer, cellulosic and non-cellulosic fiber reinforced epoxy composites. Polym. Adv. Technol..

[5] Lutz T.M., Kimna C., Casini A., Lieleg O. (2022). Bio-based and bio-inspired adhesives from animals and plants for biomedical applications. Mater. Today Bio.

[6] Heinrich L.A. (2019). Future opportunities for bio-based adhesive—Advantages beyond renewability. Green Chem..

[7] Patel A.K., Mathias J., Michaud P. (2013). Polysaccharides as Adhesives. Rev. Adhes. Adhes..

[8] Jimenez Bartolome M., Schwaiger N., Flicker R., Seidl B., Kozich M., Nyanhongo G.S., Guebitz G.M. (2022). Enzymatic synthesis of wet-resistant lignosulfonate-starch adhesives. New Biotechnol..

[9] Jimenez Bartolome M., Padhi S.S.P., Fichtberger O.G., Schwaiger N., Seidl B., Kozich M., Nyanhongo G.S., Guebitz G.M. (2022). Improving Properties of Starch-Based Adhesives with Carboxylic Acids and Enzymatically Polymerized Lignosulfonates. Int. J. Mol. Sci..

[10] Henn A.K., Forsell S., Pietiläinen A., Forsman N., Smal I., Nousaianen P., Ashok R.B.B., Oinas P., Österberg M. (2022). Interfacial catalysis and lignin nanoparticles for strong fire- and water-resistant composites adhesives. Green Chem..

[11] Yamada K., Chen T., Kumar G., Vesnovsky O., Topoleski L.D.T., Payne G.F. (2000). Chitosan Based Water-Resistant Adhesive. Analogy to Mussel Glue. Biomacromolecules.

[12] Mati-Baouche N., Elchinger P., de Baynast H., Pierre G., Delattre C., Michaud P. (2014). Chitosan as an adhesive. Eur. Polym. J..

[13] Aranaz I., Alcántara A.R., Civera M.C., Arias C., Elorza B., Heras Caballero A., Acosta N. (2021). Chitosan: An Overview of Its Properties and Applications. Polymers.

[14] Kidibule P.E., Costa J., Atrei A., Plou F.J., Fernandez-Lobato M., Pogni R. (2021). Production and characterization of chitooligosaccharides by the fungal chitinase Chit42 immobilized on magnetic nanoparticles and chitosan beads: Selectivity, specificity and improved operational utility. RSC Adv..

[15] Morin-Crini N., Lichtfouse E., Torri G., Crini G. (2019). Applications of chitosan in food, pharmaceuticals, medicine, cosmetics, agriculture, textiles, pulp and paper, biotechnology, and environmental chemistry. Environ. Chem. Lett..

[16] Alavarse A.C., Frachini E.C.G., Da Silva R.L.C.G., Lima V.H., Shavandi A., Petri D.F.S. (2022). Crosslinkers for polysaccharides and proteins: Synthesis conditions, mechanisms, and crosslinking efficiency, a review. Int. J. Biol. Macromol..

[17] Silvestre J., Delattre C., Michaud P., de Baynast H. (2021). Optimization of Chitosan Properties with the Aim of a Water Resistant Adhesive Development. Polymers.

[18] Silverman H.G., Roberto F.F. (2007). Understanding Marine Mussel Adhesion. Mar. Biotechnol..

[19] Sun P., Mei S., Xu J.F., Zhang X. (2022). A Bio-Based Supramolecular Adhesive: Ultra-High Adhesion Strengths at both Ambient and Cryogenic Temperatures andExcellent Multi-Reusability. Adv. Sci..

[20] Al Khatib M., Harir M., Costa J., Baratto M.C., Schiavo I., Trabalzini L., Pollini S., Rossolini G.M., Basosi R., Pogni R. (2018). Spectroscopic Characterization of Natural Melanin from a Streptomyces cyaneofuscatus Strain and Comparison with Melanin Enzymatically Synthesized by Tyrosinase and Laccase. Molecules.

[21] Tadyszak K., Mrówczyński R., Carmieli R. (2021). Electron Spin Relaxation Studies of Polydopamine Radicals. J. Phys. Chem. B.

[22] Al Khatib M., Costa J., Baratto M.C., Basosi R., Pogni R. (2020). Paramagnetism and Relaxation Dynamics in Melanin Biomaterials. J. Phys. Chem. B.

[23] Al Khatib M., Costa J., Spinelli D., Capecchi E., Saladino R., Baratto M.C., Pogni R. (2021). Homogentisic Acid and Gentisic Acid Biosynthesized Pyomelanin Mimics: Structural Characterization and Antioxidant Activity. Int. J. Mol. Sci..

[24] Malucelli G. (2020). Flame-Retardant Systems Based on Chitosan and Its Derivatives: State of the Art and Perspectives. Molecules.

[25] Heras-Saizarbitoria I. (2018). ISO 9001, ISO 14001, and New Management Standards.

[26] (2009). Adhesives—Determination of Tensile Lap-Shear Strength of Bonded Assemblies—Klebstoffe.

[27] Ryu J.H., Hong S., Lee H. (2015). Bio-inspired adhesive catechol-conjugated chitosan for biomedical applications: A mini review. Acta Biomater..

[28] Samyn P. (2021). A platform for functionalization of cellulose, chitin/chitosan, alginate with polydopamine: A review on fundamentals and technical applications. Int. J. Biol. Macromol..

[29] Li F., Yu Y., Wang Q., Yuan J., Wang P., Fan X. (2018). Polymerization of dopamine catalyzed by laccase: Comparison of enzymatic and conventional methods. Enzym. Microb. Technol..

[30] Miron A., Sarbu A., Zaharia A., Sandu T., Iovu H., Fierascu R.C., Neagu A., Chiriac A., Iordache T. (2022). A Top-Down Procedure for Synthesizing Calcium Carbonate-Enriched Chitosan from Shrimp Shell Wastes. Gels.

[31] Drabczyk A., Kudłacik-Kramarczyk S., Głąb M., Kędzierska M., Jaromin A., Mierzwiński D., Tyliszczak B. (2020). Physicochemical Investigations of Chitosan-Based Hydrogels Containing Aloe Vera Designed for Biomedical Use. Materials.

[32] Cheng W., Fan F., Zhang Y., Pei Z., Wang W., Pei Y. (2017). A Facile Approach for Fabrication of Core-Shell Magnetic Molecularly Imprinted Nanospheres towards Hypericin. Polymers.

[33] Khosravi H., Naderi R., Ramezanzadeh B. (2022). Synthesis and application of molybdate-doped mussel-inspired polydopamine (MI-PDA) biopolymer as an effective sustainable anti-corrosion substance for mild steel in NaCl solution. Biomass Conversion and Biorefinery.

[34] Mehwish N., Xu M., Zaeem M., Lee B.H. (2022). Mussel-Inspired Surface Functionalization of Porous Albumin Cryogels Supporting Synergistic Antibacterial/Antioxidant Activity and Bone-Like Apatite Formation. Gels.

[35] Liu Y., Zhang Z., Lv H., Qin Y., Deng L. (2018). Surface modification of chitosan film via polydopamine coating to promote biomineralization in bone tissue engineering. J. Bioact. Compat. Polym..

[36] Capitain C., Wagner S., Hummel J., Tippkötter N. (2021). Investigation of C–N Formation Between Catechols and Chitosan for the Formation of a Strong, Novel Adhesive Mimicking Mussel Adhesion. Waste Biomass Valorization.

[37] Mucha M. (1997). Rheological characteristics of semi-dilute chitosan solutions. Macro Chem. Phys..

[38] Patel A.K., Michaud P., De Baynast H., Grédiac M., Mathias J. (2012). Preparation of chitosan-based adhesives and assessment of their mechanical properties. J. Appl. Polym. Sci..

[39] Berger J., Reist M., Mayer J.M., Felt O., Peppas N.A., Gurny R. (2004). Structure and interactions in covalently and ionically crosslinked chitosan hydrogels for biomedical applications. Eur. J. Pharm. Biopharm..

[40] Cheng X., Guan J., Yang X., Tang R., Yao F. (2019). A bio-resourced phytic acid/chitosan polyelectrolyte complex for the flame retardant treatment of wool fabric. J. Clean. Prod..

[41] Zhu W., Hao S., Yang M., Cheng B., Zhang J. (2020). A synergistic flame retardant of glycosyl cross-linking boron acid and ammonium salt of phytic acid to enhance durable flame retardancy of cotton fabrics. Cellulose.

[42] Ciro Y., Rojas J., Di Virgilio A.L., Alhajj M.J., Carabali G.A., Salamanca C.H. (2020). Production, physicochemical characterization, and anticancer activity of methotrexate-loaded phytic acid-chitosan nanoparticles on HT-29 human colon adenocarcinoma cells. Carbohydr. Polym..

[43] Kaloti M., Bohidar H.B. (2010). Kinetics of coacervation transition versus nanoparticle formation in chitosan–sodium tripolyphosphate solutions. Colloids Surf. B Biointerfaces.

[44] Sharmin N., Rosnes J.T., Prabhu L., Böcker U., Sivertsvik M. (2022). Effect of Citric Acid Cross Linking on the Mechanical, Rheological and Barrier Properties of Chitosan. Molecules.

[45] Muzzarelli R.A.A., El Mehtedi M., Bottegoni C., Aquili A., Gigante A. (2015). Genipin-Crosslinked Chitosan Gels and Scaffolds for Tissue Engineering and Regeneration of Cartilage and Bone. Mar. Drugs.

[46] Beppu M.M., Vieira R.S., Aimoli C.G., Santana C.C. (2007). Crosslinking of chitosan membranes using glutaraldehyde: Effect on ion permeability and water absorption. J. Membr. Sci..

[47] Pavoni J.M.F., dos Santos N.Z., May I.C., Pollo L.D., Tessaro I.C. (2021). Impact of acid type and glutaraldehyde crosslinking in the physicochemical and mechanical properties and biodegradability of chitosan films. Polym. Bull..

[48] Kildeeva N., Chalykh A., Belokon M., Petrova T., Matveev V., Svidchenko E., Surin N., Sazhnev N. (2020). Influence of Genipin Crosslinking on the Properties of Chitosan-Based Films. Polymers.

[49] Argin-Soysal S., Kofinas P., Lo Y.M. (2009). Effect of complexation conditions on xanthan–chitosan polyelectrolyte complex gels. Food Hydrocoll..

[50] Shibaev A.V., Muravlev D.A., Muravleva A.K., Matveev V.V., Chalykh A.E., Philippova O.E. (2020). pH-Dependent Gelation of a Stiff Anionic Polysaccharide in the Presence of Metal Ions. Polymers.

[51] Lawall Werneck Cerqueira A.F., Protta Neiva G., Fernandes M.F., Leira Mota Conegundes J., Stephani R., Cappa de Oliveira L.F., da Costa Ludwig Z.M., de Carvalho dos Anjos V., Pinto Vilela F.M., Scio E. (2020). Influence of the xanthan gum as a crosslinking agent on the physicochemical properties of chitosan microparticles containing green coffee extract. Biocatal. Agric. Biotechnol..

[52] Paiva D., Gonçalves C., Vale I., Bastos M.M.S.M., Magalhães F.D. (2016). Oxidized Xanthan Gum and Chitosan as Natural Adhesives for Cork. Polymers.

