# Supplementary material for: A Novel Bio-Adhesive Based on Chitosan-Polydopamine-Xanthan Gum for Glass, Cardboard and Textile Commodities
Source: Polymers (Basel). 2024 Jun 26;16(13):1806. doi: 10.3390/polym16131806 (PMC11244100; doi:10.3390/polym16131806)
Supplement: Supplementary file 1 [file polymers-16-01806-s001.zip › polymers-3048940-supplementary.pdf]

Supplementary Materials

# A Novel Bio-Adhesive Based on Chitosan-Polydopamine-Xanthan Gum for Glass, Cardboard and Textile Commodities

**Jessica Costa [1,2], Maria Camilla Baratto [1,2], Daniele Spinelli [3], Gemma Leone [1,2], Agnese Magnani [1,2] and Rebecca Pogni [1,2,*]**

[1] Department of Biotechnology, Chemistry and Pharmacy, University of Siena, Via Aldo Moro 2, 53100 Siena, Italy; jessica.costa2@unisi.it (J.C.); mariacamilla.baratto@unisi.it (M.C.B.); gemma.leone@unisi.it (G.L.); agnese.magnani@unisi.it (A.M.)
[2] Centre for Colloid and Surface Science (CSGI), Via della Lastruccia 3, 50019 Sesto Fiorentino, Italy
[3] Next Technology Tecnotessile, Via del Gelso 13, 59100 Prato, Italy; daniele.spinelli@tecnotex.it
* Correspondence: rebecca.pogni@unisi.it; Tel.: +39-0577232120

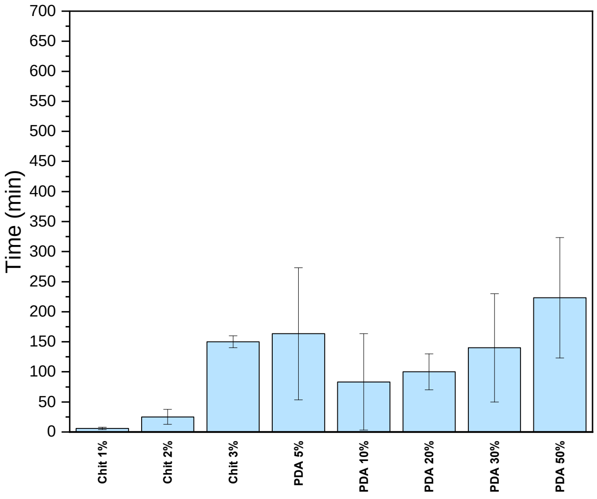


**Figure S1.** Water Resistance Analysis: Different concentrations of chitosan solution (1%, 2%, and 3%). The chitosan solution (3%) was selected, and various concentrations of polydopamine (5%, 10%, 30%, and 50% - relative to the amount of chitosan) were added. All samples were tested in triplicate.

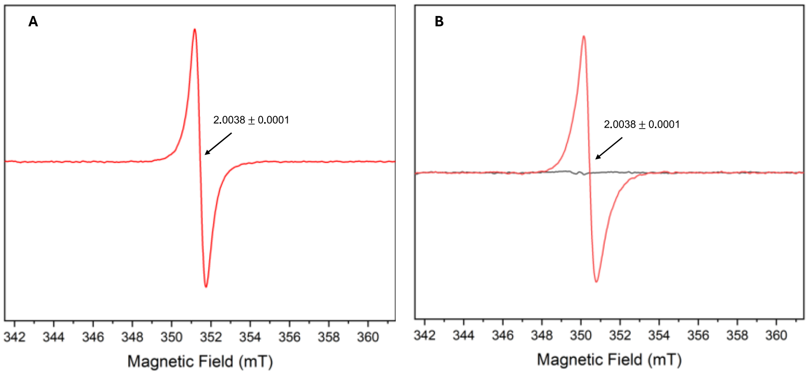


**Figure S2**. Continuous Wave Electron Paramagnetic Resonance Spectroscopy (CW-EPR) measurements at X-band (n = 9.87 GHz). (**A**) Polydopamine spectrum (**B**) Adhesive formulation in textile. black line: textile

without adhesive, red line: textile covered by chitosan/polydopamine adhesive. The analysis was performed at room temperature.

**Table S1**. Results of mechanical tests performed on two commercial glues

| | | Section (mm²) | Fmax (N) | Tensile strength *(N/mm²) |
|---|---|---|---|---|
| **Cardboard** | Uhu glue stick | 800 | 362.4 | 0.453 |
| | Hot glue Tiger | - | n.d | n.d. |
| **Textile** | Uhu glue stick | 900 | 142.4 | 0.158 |
| | Hot glue Tiger | 500 | 125.8 | 0.252 |

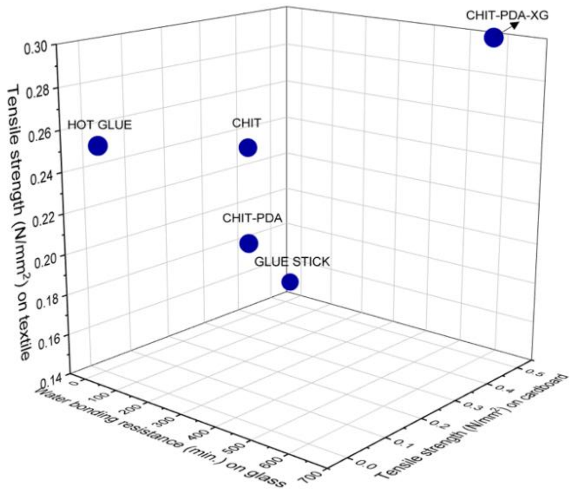


**Figure S3.** Investigation of adhesive formulations on their performance onto glass (water bonding resistance), cardboard and textile (tensile strength). The samples compared include: 3% chitosan, 3% chitosan with 50% extrinsic polydopamine, 3% chitosan with 50% extrinsic polydopamine and 3% xanthan gum, commercial glue stick, and commercial hot glue

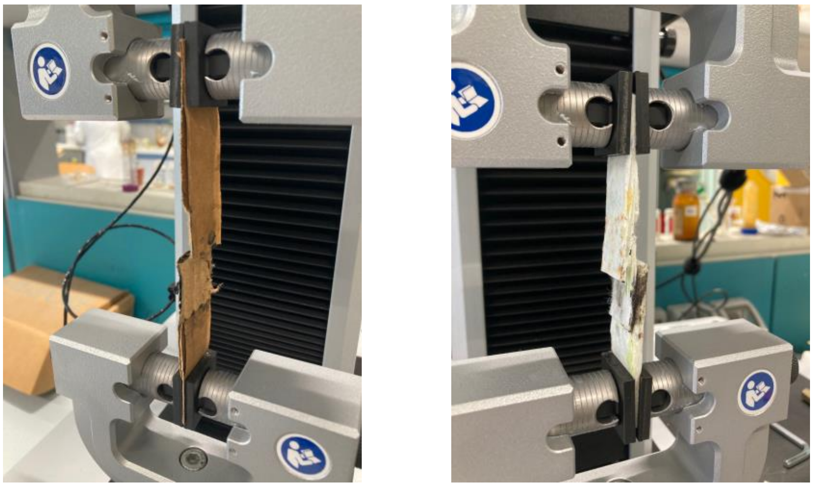

**Figure S4.** Photos taken during the mechanical testing of the chitosan/polydopamine/xanthan gum adhesive in cardboard (on the left) and textile (on the right).
